# Use of Levosimendan in Cardiac Surgery: An Update After the LEVO-CTS, CHEETAH, and LICORN Trials in the Light of Clinical Practice

**DOI:** 10.1097/FJC.0000000000000551

**Published:** 2017-10-30

**Authors:** Fabio Guarracino, Matthias Heringlake, Bernard Cholley, Dominique Bettex, Stefaan Bouchez, Vladimir V. Lomivorotov, Angela Rajek, Matti Kivikko, Piero Pollesello

**Affiliations:** * Dipartimento di Anestesia e Terapie Intensive, Azienda Ospedaliero-Universitaria Pisana, Pisa, Italy;; † Klinik für Anästhesiologie und Intensivmedizin, Universitätsklinikum Schleswig-Holstein, Lübeck, Germany;; ‡ Department of Anesthesiology and Critical Care Medicine, Hôpital Européen Georges Pompidou, Paris, France;; § Université Paris Descartes, Sorbonne Paris Cité, Paris, France;; ¶ Cardiac Anaesthesia, University Hospital, Zurich, Switzerland;; ‖ Department of Anesthesiology, University Hospital, Ghent, Belgium;; ** Department of Anesthesiology, E. Meshalkin National Medical Research Center, Novosibirsk, Russia;; †† Klinische Abteilung für Herz-Thorax-Gefäßchirurgische Anästhesie und Intensivmedizin, Medical University of Vienna, Vienna, Austria;; ‡‡ Critical Care Proprietary Products, Orion Pharma, Espoo, Finland; and; §§ Department of Cardiology S7, Jorvi Hospital, Espoo, Finland.

**Keywords:** cardiac surgery, clinical trials, levosimendan, systematic review, opinion paper

## Abstract

Levosimendan is a calcium sensitizer and adenosine triphosphate–dependent potassium channel opener, which exerts sustained hemodynamic, symptomatic, and organ-protective effects. It is registered for the treatment of acute heart failure, and when inotropic support is considered appropriate. In the past 15 years, levosimendan has been widely used in clinical practice and has also been tested in clinical trials to stabilize at-risk patients undergoing cardiac surgery. Recently, 3 randomized, placebo-controlled, multicenter studies (LICORN, CHEETAH, and LEVO-CTS) have been published reporting on the perioperative use of levosimendan in patients with compromised cardiac ventricular function. Taken together, many smaller trials conducted in the past suggested beneficial outcomes with levosimendan in perioperative settings. By contrast, the latest 3 studies were neutral or inconclusive. To understand the reasons for such dissimilarity, a group of experts from Austria, Belgium, Finland, France, Germany, Italy, Switzerland, and Russia, including investigators from the 3 most recent studies, met to discuss the study results in the light of both the previous literature and current clinical practice. Despite the fact that the null hypothesis could not be ruled out in the recent multicenter trials, we conclude that levosimendan can still be viewed as a safe and effective inodilator in cardiac surgery.

## INTRODUCTION

Perioperative mortality is reported to be as low as 1%–4% in the general elective surgery population.^1^ However, in patients with postoperative low cardiac output syndrome (LCOS), mortality is considerably higher.^2^ In addition to higher mortality, LCOS predisposes patients to postoperative myocardial injury, renal failure, and prolonged intensive care unit (ICU) and hospital stay.^3^ Several baseline factors, such as preoperatively reduced left (and/or right) ventricular function or recent myocardial infarction, predispose patients to LCOS.^4^ The type of surgery also affects the postoperative risk profile; coronary artery bypass grafting (CABG) alone has a more benign outcome than, for example, the combination of CABG and a valve replacement.^5^

LCOS is managed with inotropic agents and/or mechanical cardiac assist devices such as an intraaortic balloon pump (IABP). Even so, short-term mortality is greatly elevated versus non-LCOS comparators.^6^ Moreover, the inotropic agents traditionally used in this setting have conspicuous adverse effects or incompletely defined safety profiles.^7^

Levosimendan is a calcium sensitizer and adenosine triphosphate (ATP)-dependent potassium channel opener with positive inotropic, vasodilatory, and cardioprotective properties.^8^ The drug binds to cardiac troponin C in a calcium-dependent manner,^9,10^ which mediates the positive inotropic effect by increasing the calcium sensitivity of myocytes. The vasodilatory effect is due to the opening of ATP-sensitive potassium channels in vascular smooth muscle, resulting in its relaxation. By opening mitochondrial ATP-sensitive potassium channels in cardiomyocytes, the drug also exerts a cardioprotective effect. In addition, inhibition of phosphodiesterase III by levosimendan has been also proposed to have a role in its pharmacodynamics effects.^11^

Levosimendan has been in clinical use for 15 years. In addition to its original indication for acutely decompensated heart failure, it has also been used to stabilize patients undergoing cardiac surgery. Abundant literature from exploratory studies supports the rationale for its use in this indication,^12^ and this is also supported by its benign effect on kidney function.^13^

Recently, 3 randomized, placebo-controlled, multicenter studies were published on the perioperative use of levosimendan: LICORN,^14^ CHEETAH,^15^ and LEVO-CTS.^16^ In contrast to the many preceding smaller trials which, either individually or as a whole, produced a promising image of levosimendan in perioperative settings, these latest 3 studies were either neutral or inconclusive.

A group of experts from 8 European countries (Austria, Belgium, Finland, France, Germany, Italy, Switzerland, and Russia), including investigators from the 3 most recent studies on the preoperative, perioperative, and postoperative use of levosimendan, met on April 20, 2017 in occasion of the EACTA annual congress in Berlin, Germany, to discuss the recent study results in the light of both the previous literature and current clinical practice. The present article was created from the proceedings of that discussion.

## PREVIOUS RELEVANT STUDIES ON THE USE OF LEVOSIMENDAN IN CARDIAC SURGERY

Levosimendan has been studied in >40 clinical trials in cardiac surgery. Earlier studies suggested that it could prevent the development of LCOS and be effective in treating postoperative LCOS (Box 1). The level of proof, however, remained low, despite a meta-analysis that suggested a survival benefit in patients with low preoperative ejection fraction (EF).^17^ Indications of renal-protective effects in this setting have also been reported in retrospective analyses.^18,19^

BOX 1. **Previous Relevant Studies on the Use of Levosimendan in Cardiac Surgery**

- Over 40 clinical trials were run on the use of levosimendan in cardiac surgery;
- Earlier studies suggested that levosimendan could prevent the development of LCOS and could be effective in treating postoperative LCOS;
- Meta-analyses suggested a reduction of mortality, significant when levosimendan is used in case of severe perioperative cardiovascular dysfunction (LVEF ≤ 30%);
- Indications of favorable renal effects in this setting have also been reported.

**FIGURE 1.**
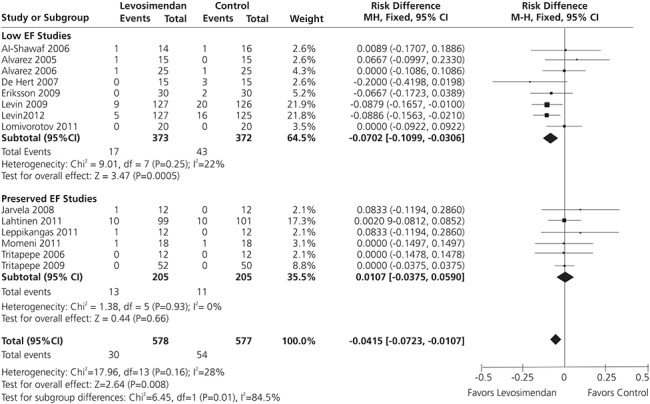
Meta-analysis of data from 14 randomized controlled trials of perioperative levosimendan in cardiac surgery patients (n = 1155) indicates that levosimendan therapy is associated with reduced mortality, with the greatest benefit observed in patients with reduced LVEF, from Harrison et al.^17^

The individual and aggregate findings of the 14 studies in cardiac surgery patients with low left ventricular EF (LVEF) examined by Harrison et al^17^ in their meta-analysis are reported in Figure 1, and the results of the contributing studies are summarized briefly in Table 1.^20–33^

**TABLE 1.**
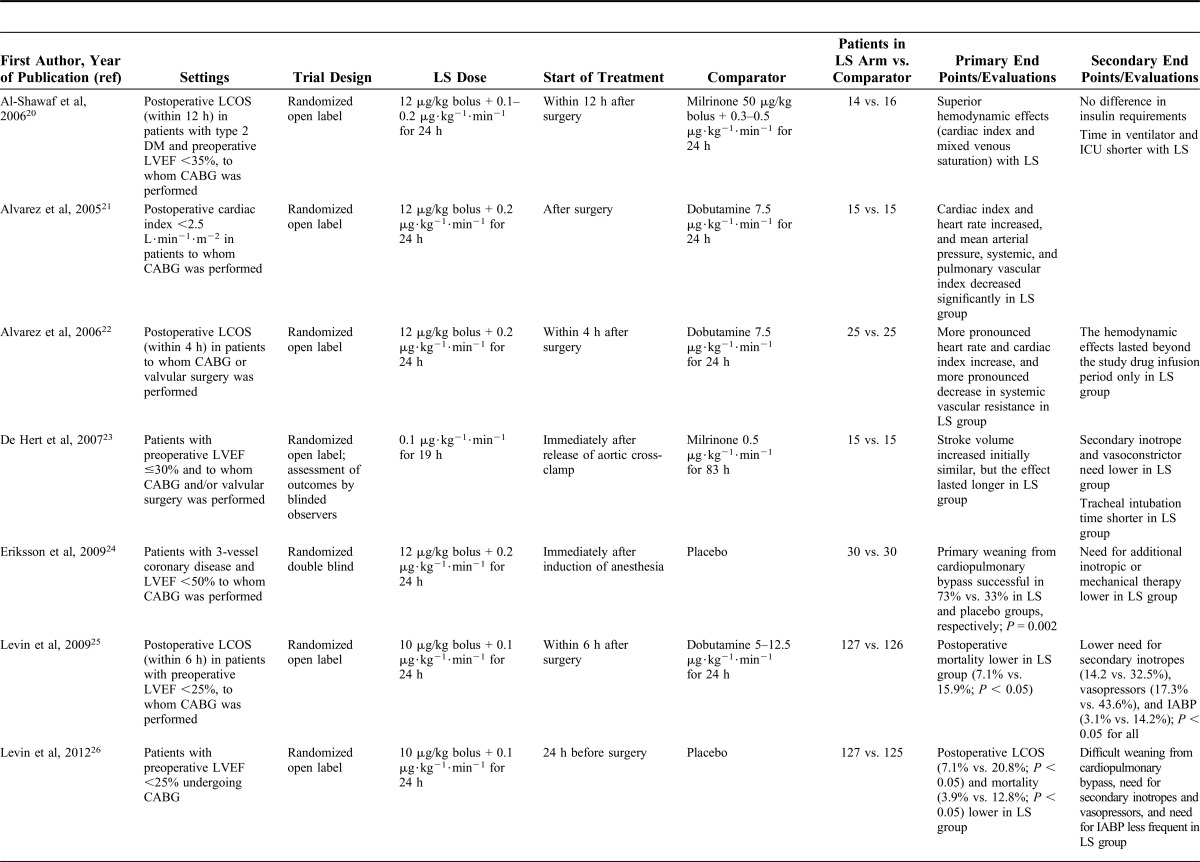

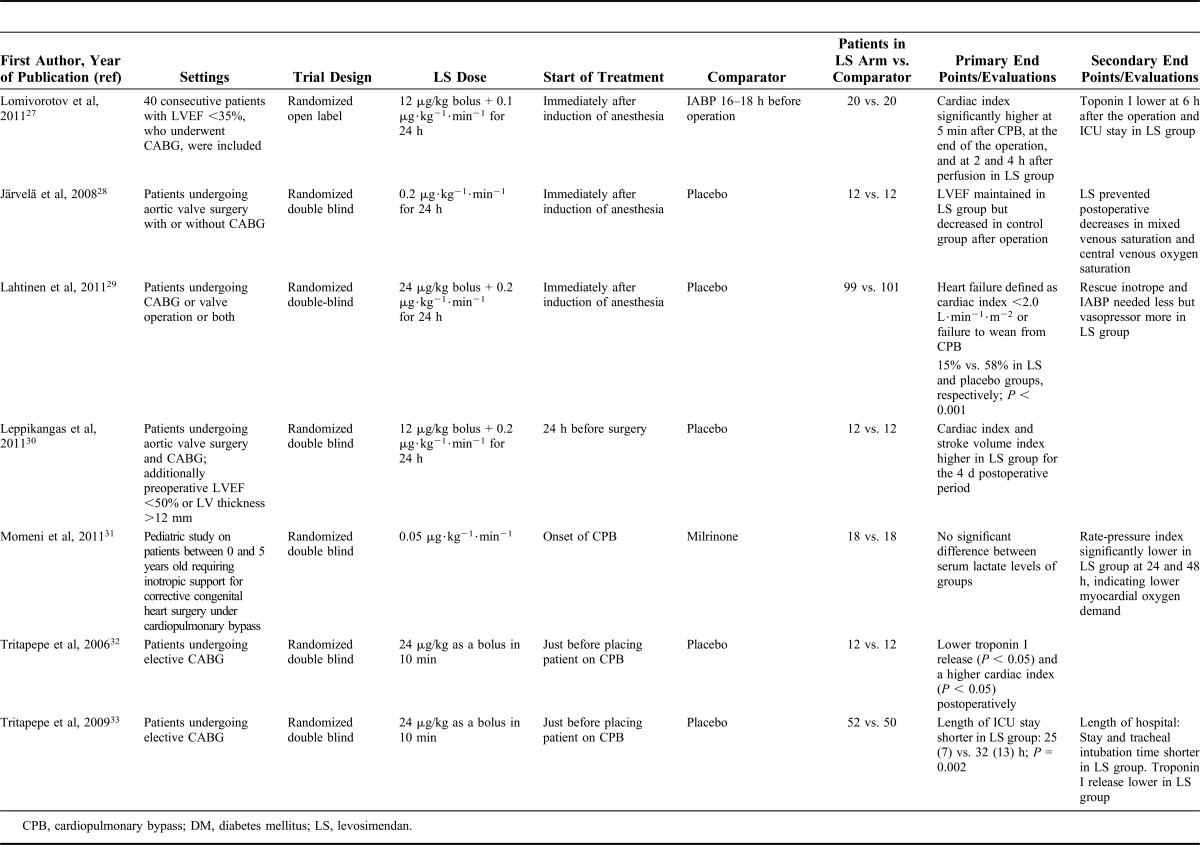
Studies on the Use of Levosimendan in Perioperative Settings Included in the Meta-analysis by Harrison et al^17^

In total, Harrison et al took into consideration data from 1155 patients (84 deaths) and the overall effect of levosimendan versus comparator was significant (*P* = 0.008). Visual inspection of the funnel plot for the primary outcome of mortality was not suggestive of significant publication bias. However, removal of either of the 2 studies by Levin et al^25,26^ made the overall estimated effect of levosimendan on mortality insignificant.

For the sake of completeness, we report that, in addition to the 14 articles considered by Harrison et al, other relevant studies conducted on levosimendan in surgical patients are those by Severi et al^34^ and Lomivorotov et al^35^ on levosimendan versus IABP; the trials by Baysal et al^36^ and Erb et al^37^ on renal outcome and organ dysfunction, respectively; and the randomized pilot study on prophylactic use of levosimendan by Anastasiadis et al.^38^

## THE 3 MOST RECENT STUDIES

Three larger clinical studies have recently been conducted with levosimendan in patients undergoing cardiac surgery. All 3 were randomized, placebo-controlled, multicenter studies. The LICORN and CHEETAH studies were investigator-initiated studies, whereas LEVO-CTS was a phase 3 regulatory study (Box 2). Broad outlines of the study designs and their primary findings are given below.

BOX 2. **The 3 Most Recent Studies on Levosimendan in Cardiac Surgery**

- LICORN: Levosimendan on Low Cardiac Output Syndrome in Patients With Low Ejection Fraction Undergoing Coronary Artery Bypass Grafting With Cardiopulmonary Bypass trial (NCT02184819) assessing the efficacy of a preoperative infusion of levosimendan in reducing postoperative LCOS in patients with poor LVEF undergoing CABG;
- CHEETAH: Levosimendan to Reduce Mortality in High Risk Cardiac Surgery Patients trial (NCT00994825) assessing the effect of levosimendan on cardiac surgery patients who developed postoperative LCOS;
- LEVO-CTS: Levosimendan in Patients with Left Ventricular Systolic Dysfunction Undergoing Cardiac Surgery Requiring Cardiopulmonary Bypass, phase III clinical trial (NCT02025621) assessing the effect of levosimendan on patients with low preoperative LVEF (EF < 35%) undergoing scheduled or urgent cardiac surgery.

### LICORN

The LICORN trial (Levosimendan on Low Cardiac Output Syndrome in Patients With Low Ejection Fraction Undergoing Coronary Artery Bypass Grafting With Cardiopulmonary Bypass; NCT02184819) assessed the efficacy of a preoperative infusion of levosimendan in reducing postoperative LCOS in patients with poor LVEF undergoing CABG.^14^ A cohort of 336 patients with LVEF ≤40% undergoing CABG was recruited from 13 French hospitals. The study drug was started after induction of anesthesia and infused over a period of 24 hours at a rate of 0.1 μg·kg^−1^·min^−1^. Postoperative LCOS was evaluated using a composite criterion comprising (1) need for inotropic agents beyond 48 hours after initiation of the study drug; (2) need for postoperative mechanical assist devices or failure to wean from these devices when inserted preoperatively; and (3) need for renal replacement therapy.

The primary end point was observed in 87/167 patients (52%) in the levosimendan group, compared with 101/168 (61%) in the placebo group [absolute risk reduction −7%, 95% confidence interval (CI), −17% to +3%, *P* = 0.15]. Of the secondary end points, the duration of catecholamine treatment was shorter in the levosimendan group: 3.2 ± 3.6 versus 4.1 ± 4.3 days (*P* = 0.021). However, no adjustment was made for multiple comparisons, and this result should be considered exploratory. There were no statistically significant intergroup differences in mortality or length of ICU stay.

The lack of statistical significance in the composite primary end point was likely contributed to by the fact that the observed event rate in the placebo arm was lower than that anticipated (61% vs. 65%); it was anticipated also that the prevalence rate would be reduced to 50% in the levosimendan group, but the prevalence actually observed was 52% in the intention-to-treat population (vs. 51% in the per-protocol population). LICORN was powered according to an expectation of an absolute risk reduction of 15%. The point estimate actually recorded was 7% and favored levosimendan, but the 95% CI included a reduction of 17% (range −17% to +3%). The observed effect was less than that anticipated by the study hypothesis; however, the study was underpowered to definitely exclude a meaningful beneficial effect of levosimendan on the primary composite outcome.

### CHEETAH

In the CHEETAH (Levosimendan to Reduce Mortality in High Risk Cardiac Surgery Patients; NCT00994825) trial, levosimendan or placebo was administered to cardiac surgery patients, who, according to predefined criteria, developed postoperative LCOS.^15^ In total, 1000 patients were scheduled to be included and the primary end point was 30-day mortality. The study was performed in 14 centers in Italy, Russia, and Brazil but was stopped for futility after 506 patients had been enrolled. A total of 248 patients received levosimendan and 258 received placebo. The mean infusion rate and duration of levosimendan were 0.07 μg·kg^−1^·min^−1^ for 33 hours, and the median EF was 50% in both groups, with 11% of patients having an EF of <25%. There was no significant difference in 30-day mortality between the levosimendan and placebo groups: 32 patients (12.9%) versus 33 (12.8%); absolute risk difference 0.1 percentage points; 95% CI, −5.7 to +5.9 percentage points; *P* = 0.97. There were no significant between-group differences in other end points and no difference in the rates of adverse events (hypotension or arrhythmias).

It should be noted that, in the report of the CHEETAH study, preparation of the study drug was described as follows^15^: “levosimendan was diluted as 12.5 mg in 100 mL of 5% glucose.” This is at variance with the summary of product characteristics guidance, according to which 1 vial of Simdax (12.5 mg levosimendan concentrate for intravenous) should be diluted in at least 250 mL of 5% glucose solution (1:50). There is a risk of precipitation if smaller diluent volumes are used and this exposes the patient to unpredictable dosing (ie, receipt of less than the intended dose).

### LEVO-CTS

This study (Levosimendan in Patients with Left Ventricular Systolic Dysfunction Undergoing Cardiac Surgery Requiring Cardiopulmonary Bypass; NCT02025621) was a phase III clinical trial sponsored by TENAX Therapeutics Inc, run by Duke University (Durham, NC) and configured to support a marketing authorization in the United States and Canada for levosimendan.^16^ The study design and objectives were discussed beforehand and agreed with the US Food and Drug Administration (FDA).

The study population consisted of 882 patients with low preoperative LVEF (EF < 35%) undergoing scheduled or urgent cardiac surgery (CABG and/or mitral valve surgery with or without involvement of other valves). All patients were considered at risk of developing postoperative LCOS. Levosimendan (0.2 μg·kg^−1^·min^−1^ for 60 minutes, followed by 0.1 μg·kg^−1^·min^−1^ for 23 hours) or placebo was started at the induction of anesthesia to assess whether the drug would decrease the development of LCOS and its detrimental consequences.

The study, conducted at 70 sites in Canada and the United States, demonstrated no statistically robust treatment effect on the composite primary end point of death, perioperative myocardial infarction, and need for renal replacement therapy or a mechanical ventricular assist device. However, there were fewer deaths in the levosimendan group: 20/428 (4.7%) versus 30/421 (7.1%), odds ratio 0.64, 95% CI, 0.37–1.13 (*P* = 0.12). In addition, the levosimendan-treated patients experienced statistically significantly fewer LCOS events (78 vs. 108; *P* = 0.007) and needed less inotropic support at or beyond 24 hours after initiation of infusion (235 vs. 264; *P* = 0.02). Cardiac index also improved more in levosimendan-treated patients (2.9 ± 0.6 vs. 2.7 ± 0.7 L·min^−1^·m^−2^; *P* < 0.001). Hypotension and atrial arrhythmias were recorded as adverse events with similar frequency in both study groups.

**FIGURE 2.**
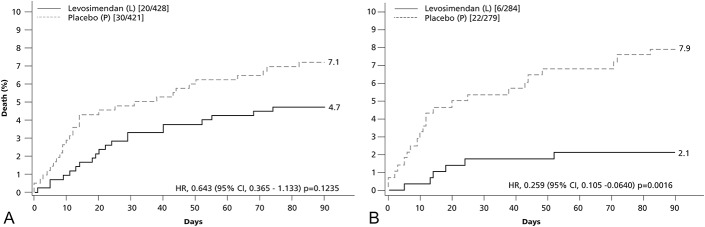
Ninety-day mortality among patients in the LEVO-CTS trial in (A) the whole study (n = 849) and (B) the subgroup of isolated CABG patients (n = 563). In the latter, mortality was significantly lower in the levosimendan arm than in the placebo arm, from supplemental materials in Mehta et al.^16^ HR, hazards ratio.

The LEVO-CTS investigators are conducting post hoc analyses in relevant predetermined subsettings (eg, CABG with or without accompanying valve surgery). So far, they have shown data suggesting that in those patients in whom only CABG was performed, mortality was significantly lower in the levosimendan group: 6/284 (2.1%) versus 22/279 (7.9%), hazard ratio 0.259, 95% CI, 0.105–0.640 (*P* = 0.0016) (Fig. 2).

## INTERPRETATION OF THE DATA

### Efficacy

The hypotheses tested in all 3 studies were not affirmed, and the primary end points did not differ significantly between the levosimendan and control arms.

It must be noted that the end points used in LEVO-CTS and LICORN were experimental ones. Similar end points have not been used in earlier studies. The LEVO-CTS primary end points were agreed with the FDA, which required the inclusion of clinical events. This notwithstanding, encouraging evidence of efficacy emerged from LEVO-CTS: the lower incidence of LCOS, lesser need for inotropic support by catecholamines, and improvement in CI indicate that levosimendan exhibited efficacy. In CHEETAH, only suggestive signs of improved hemodynamics were noted, but the dose of levosimendan in that study was smaller than those in LEVO-CTS or LICORN or in previous studies in cardiac surgery. The subgroup data from LEVO-CTS (the supplementary material of the publication is available through http://www.nejm.org/doi/full/10.1056/NEJMoa1616218) suggest that levosimendan may be more effective in patients in whom only the CABG procedure is performed (and ineffective in valve replacement patients). Also, in line with earlier data, patients with the lowest EF before surgery may benefit most from the treatment. However, this was not observed in the LICORN study, in which no statistically significant difference was found with respect to the primary end point between patients with LVEF <30% or between 30% and 40%.

**FIGURE 3.**
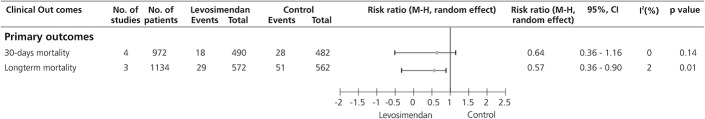
Meta-analysis of clinical trials on levosimendan versus control in cardiac surgery patients with long-term and thirty-day mortality as primary outcome: Effects of levosimendan when used in case of severe perioperative cardiovascular dysfunction (LVEF ≤ 30%). Sensitivity analysis as in the supplemental material of Lee et al.^47^

Although the duration of treatment with inotropic agents was an outcome variable, the LICORN and CHEETAH protocols do not give details of hemodynamic monitoring and specifically when inotrope treatment should be stopped. Regarding the limited efficacy of levosimendan, the regimen used in the trials can be questioned. Both LEVO-CTS and LICORN used a 0.1 μg·kg^−1^·min^−1^ infusion for 24 hours without bolus, in conformity with recent recommendations by experts in cardiovascular anesthesia.^12^ Several previous studies have used infusion rates of 0.2 μg·kg^−1^·min^−1^ with variable bolus doses. Higher doses may have produced greater hemodynamic effects but at the expense of a more potent vasodilatation and consequent hypotension.

In the LICORN trial, inodilators and inopressors were analyzed together as “catecholamines” irrespective of their predominant hemodynamic effect and no doses for the respective drugs were reported. Consequently, it cannot be ruled out that patients with a severe postoperative LCOS receiving high doses of epinephrine and milrinone, and those needing only small doses of norepinephrine on the second day after surgery were both classified as “catecholamine-dependent.”

One additional observation regarding LEVO-CTS and LICORN is that levosimendan therapy was started very shortly before surgery; accordingly, there was only a short time during which levosimendan could exert any preconditioning effect. In some previous studies, and in clinical practice, levosimendan has been administered for up to 24 hours before the start of surgery.^26,30^

Finally, in the CHEETAH trial, most patients received a relatively low dose of levosimendan while already being treated with high doses of epinephrine and dobutamine. Pretreatment with beta-mimetic drugs has been shown to reduce the inotropic effect of levosimendan in vitro^39^ and thereby may also reduce its benefits in vivo, as shown by Bonios et al^40^ in a trial comparing the event-free survival of patients treated with levosimendan, dobutamine, or their combination.

### Safety

Safety was not identified as a concern in any of these 3 studies: there was no significant excess of arrhythmias or hypotension and no increase in mortality in levosimendan-treated patients. In fact, mortality was numerically lower in levosimendan-treated patients in LEVO-CTS.^16^

These findings are fully consistent with the data on safety and adverse events reported in most of the previous studies (as collected in a systematic review and meta-analysis by Landoni et al^41^) and confirm levosimendan as the safest agent among the family of inotropes and inodilators which include, among others, dobutamine and milrinone.^42^ When an inotrope is needed, the safety profile of the chosen agent should be an important selection criterion.

In addition, levosimendan has a unique mechanism of action and pharmacokinetics. The sensitization of myofilaments to calcium supposes that the inotropic effects occur without (or with minimal) increase in myocardial oxygen consumption. The prolonged effect which lasts for several days,^23,43^ contrasts with the on–off action of dobutamine. These specificities may prove useful in selected patients and are a major reason to keep levosimendan in the armamentarium of physicians in charge of patients with cardiac dysfunction.

## DISCUSSION

Although the many smaller trials conducted in the past produced as a whole a promising image of levosimendan in perioperative settings, the data from the latest (and larger) 3 studies did not support the hypotheses tested.

Instead of advocating the “small-study effect,” that is, the trend for smaller studies to show larger treatment effects,^44^ every study should be evaluated fairly, as large studies can be imprecise, just as small ones can be precise.^45^

Indeed, in small monocentric trials focused on a few end points in very specific clinical settings, the researchers are in a more controlled situation than in a multicenter trial in the field. In addition, in clinical settings, where there are local variations in therapeutic approaches and tailored strategies (such as, for example, in mitral valve surgery^46^), multicenter trials add, by definition, statistical noise to many end points: variations in pharmacologic and nonpharmacologic parameters have the potential to impair the statistical power and obscure meaningful effects. Multicenter studies in fields such as perioperative LCOS, where multiple sources of heterogeneity exist (eg, symptoms, etiologies, comorbidities, comedications, and center-specific treatment practices), encounter such problems.

All the above notwithstanding, we have noticed that studies of all sizes on levosimendan have produced some common and consistent findings in terms of safety. Levosimendan is safe and well tolerated in patients undergoing cardiac surgery with cardiopulmonary bypass who have low LVEF and are at risk of the development of postoperative LCOS. This safety finding, and especially the lack of any deterioration in survival, is particularly noteworthy given that many of the patients in all 3 trials had already been treated with a range of other pressor and/or inotropic drugs. The nonattainment of the study hypotheses in these 3 recent trials does not rule out the fact that levosimendan might be effective in selected patients undergoing cardiac surgery. The LEVO-CTS study, as the largest of these trials, confirms that a prophylactic infusion of levosimendan started immediately before surgery reduces LCOS in a heterogeneous population of cardiac surgery patients with reduced LVEF. The post hoc analyses further suggest that this drug may be especially useful in patients undergoing CABG with reduced LVEF but not in those undergoing a valve surgery. A recent meta-analysis, including also the latest trials, confirms that levosimendan had a significant effect on mortality only when used in case of severe perioperative cardiovascular dysfunction (LVEF ≤ 30%) in patients receiving cardiac surgery (Fig. 3).^47^ No comparable data are available for any other drugs with inotropic properties; on the contrary, traditional inotropes are considered to have detrimental effects on outcome.^42^

In addition, levosimendan has been shown to reduce elevated right-sided pressures in various clinical situations.^48–50^ Preoperative administration of levosimendan decreased pulmonary artery pressure significantly in patients with right ventricular dysfunction and pulmonary hypertension.^51^ As pulmonary hypertension is an important prognostic factor in cardiac surgery associated with increased morbidity and mortality,^52^ levosimendan's efficacy could be pronounced in this subgroup and we warmly suggest to explore this setting.

Finally, it must be registered that in many studies, including relatively large regulatory clinical trials, levosimendan was administered in addition to standard of care (ie, other vasoactive drugs) according to prevailing practice at individual study centers. It would be instructive to perform a post hoc analysis of those data to explore whether combinations of levosimendan with dobutamine or norepinephrine are beneficial.

## CONCLUSIONS

Taking all the available data into consideration, including the experience of the 3 most recent studies, our conclusion is that levosimendan is a safe and effective agent for the treatment of patients undergoing cardiac surgery and in need for inotropic support (Box 3). However, the magnitude of effect of this agent is not as large as previously thought and 3 large multicenter trials could not rule out their null hypothesis. For this reason, levosimendan cannot be at the moment recommended for routine use in all cardiac surgery settings. Further in-depth assessment of the utility of levosimendan will require additional trials in closely defined patient populations with study designs that mitigate, to the fullest extent possible, any influence of methodological variations in patient management.

BOX 3. **Consensus on Efficacy and Safety of Levosimendan in Operative Settings**

Taking all the available data into consideration, including the experience of these 3 recent studies:

- Levosimendan is a safe agent for the treatment of patients undergoing cardiac surgery and in need for inotropic support, despite the 3 large multicenter trials could not rule out their null hypothesis;
- Levosimendan is an effective agent, as it regards hemodynamic support;
- Statistically significant mortality benefit seems to be limited to subgroups, such as the isolated CABG procedures, and the low-EF patients;
- Further in-depth assessment of the utility of levosimendan will require additional trials in closely defined patient populations with study designs that mitigate to the fullest extent as possible any influence of methodological variation.
